# Editorial: Advances in malignant pleural mesothelioma: Diagnosis, treatment, and molecular mechanisms

**DOI:** 10.3389/fonc.2023.1158416

**Published:** 2023-02-16

**Authors:** Maria Giovanna Mastromarino, Vittorio Aprile, Marco Lucchi

**Affiliations:** ^1^Division of Thoracic Surgery, Cardiac, Thoracic and Vascular Department; Pisa University Hospital, Pisa, Italy; ^2^Department of Surgical, Medical and Molecular Pathology and Critical Care Medicine, University of Pisa, Pisa, Italy

**Keywords:** malignant pleural mesothelioma, diagnosis, treatment, molecular mechanisms, multimodality strategy, precision oncology

Malignant pleural mesothelioma (MPM) represents a death sentence, with an estimated survival of less than one year after diagnosis in most cases (1). The new millennium has witnessed an MPM diagnosis outbreak due to the intensive past use of asbestos. A lack of knowledge of the pathogenesis and different prognostic aspects, together with its high socioeconomic cost, have forced research on this disease in the last decade. Nevertheless, the diagnostic modalities and treatment strategies of MPM are still far from being standardized, and the molecular mechanisms continue to be unclear.

Recently, considerable progress in molecular and histopathological analysis has led to a necessary update of the WHO Classification of Tumors of the Pleura (2). The substantial changes include the pathology revision of the latest classification system in order to incorporate architectural patterns and stromal and cytologic features as well as nuclear grading for epithelioid diffuse MPM and the molecular landscape of MPM. Particular attention has been reserved for the recognition of mesothelioma *in situ* as a precisely defined clinicopathologic entity, requiring a demonstration of loss of BAP1 and/or MTAP by immunohistochemistry and/or CDKN2A (p16) homozygous deletion by fluorescence *in situ* hybridization for differential diagnosis from reactive mesothelial proliferation (3). Despite all the advances achieved, a proper diagnosis of MPM still represents a challenge for the physician, hence the development of different biomarkers that could be useful to increase diagnosis accuracy and the efficiency of prognosis by assessing a more accurate patient risk stratification (4). In this scenario, new prognostic biomarkers as well as new potential molecular targets are strongly required to understand the molecular mechanisms of MPM and drive more efficient therapies.

To date, there is no universal standardization of therapeutic options since most patients have a late diagnosis, usually with advanced disease. In these cases, locoregional therapies gave way to systemic ones in which platinum-based combinations, with or without pemetrexed, represent the most common doublet despite yielding poor long-term outcomes (5).

Recently, immune checkpoint inhibitors have demonstrated promising activity for the treatment of MPM and have been incorporated into some treatment regimens (6). Surgery represents an effective but seriously detrimental alternative that should be reserved for selected patients. In very selected cases, multimodality approaches, including surgical resection by either extra-pleural pneumonectomy or pleurectomy-decortication after neoadjuvant chemotherapy (CHT) or followed by adjuvant CHT and/or radiotherapy represent, to date, the best alternative that may be offered to these patients (7, 8). The main limitation of surgery for MPM, in fact, is the high locoregional relapse rate that reaches 75%, due to the impossibility of achieving a radical disease-free margins resection (R0) because of the laminar tumor growth (9). This critical success-limiting factor has encouraged further research into intracavitary therapies, such as hyperthermic intrathoracic chemotherapy, to improve locoregional control by shrinking microscopic residual foci (R1 margins) more effectively (10).

Finally, the role of the tumor microenvironment (TIME) and the identification of potential biomarkers of activity/resistance to novel treatment strategies is currently a field of active study to enhance anti-tumor immunity by investigating the interaction of the tumor cells with the stroma and the surrounding host niche (11).

Recently, we collaborated on a special series on advances in the fields of diagnosis, treatment, and molecular mechanisms of MPM.

Herein, Xu et al. review the current knowledge about vulnerabilities according to functional loss of major tumor suppressor genes and dependencies evolving out of cancer development and resistance to cisplatin-based chemotherapy, with the aim to elucidate the therapeutic landscape and promote precision oncology for MPM. Lauk et al. show preliminary results of the safety and oncologic efficacy of the addition of bevacizumab to standard induction chemotherapy prior to MPM surgery, demonstrating a significant improvement in response rates without increased intra- and postoperative bleeding complications. Tostes et al. describe the first case of complete pathological response obtained after neoadjuvant chemo-immunotherapy, with the sustained benefit for the patient of being disease and treatment free up to 14 months after surgery. In a multi-center national study, Dudnik et al. test the role of BAP-1 alterations in MPM patients regarding the outcomes of systemic treatments; they conclude that BAP1-altered MPM, as compared to non-selected MPM, is characterized by similar efficacy of standard platinum-based chemotherapy and immune checkpoint inhibitors, while no responses were observed with poly (ADP-ribose) polymerase inhibitors. Duan et al. perform a combined analysis of RNA-sequence and microarray data; authors were able to establish and validate the role of the competing endogenous RNA network as a novel prognostic and therapeutic biomarker of MPM. Another potential diagnostic and prognostic marker has been identified by Guo et al., who reveals a high sensitivity and specificity for Aurora Kinase A (AURKA gene encode, Aurora-A) searching from the Gene Expression Omnibus (GEO) database. The microarray dataset from the GEO database has also been used by Endo et al. to demonstrate the role of insulin-like growth factor 2 mRNA binding protein 3 (IGF2BP3) as one of the significantly upregulated genes in MPM, which might promote cell proliferation, a critical step in oncogenesis, by suppressing the expression of p27 in malignant mesothelioma cells. Ollila et al. study the effect of the tumor immune microenvironment in epithelioid MPM, revealing its prognostic value, while Janssens et al. describe headspace volatile organic compounds (VOCs) capable of distinguishing between MPM and lung cancer cells, as well as between the histological subtypes within MPM (epithelioid, sarcomatoid and biphasic), suggesting a useful role of VOCs in generating a clinically predictive breath model for MPM. Finally, Choi et al. summarize the current state of intraoperative intrapleural therapeutic agents, providing an updated review on pleural-directed adjuncts in the management of MPM as well as highlighting the most promising near-term technology breakthroughs.

Discovering ways and strategies to overcome diagnostic challenges and limited treatment options in MPM is a constantly evolving research field. The comprehension of the molecular mechanisms in tumor development and the biomolecular landscape of MPM might pave the way for new therapeutic strategies. The study of TIME is pivotal in identifying appropriate prognostic and predictive tissue biomarkers, attempting to detect the subgroups of patients who will benefit the most from multimodality approaches. The collective goal of this scientific endeavor will be to implement personalized treatment based on the specific MPM molecular features for each patient, thus promoting precision oncology.

In conclusion, the articles in the present Research Topic provide the reader with new and ongoing research in MPM, review current management strategies and updates, and encourage further contributions in this field to improve the life and prognosis of patients suffering from such a dismal cancer.

## Author contributions

All authors listed have made a substantial, direct, and intellectual contribution to the work and approved it for publication.
